# Occupation-specific recruitment: An empirical investigation on job seekers’ occupational (non-)fit, employer image, and employer attractiveness

**DOI:** 10.3389/fpsyg.2022.937116

**Published:** 2022-09-23

**Authors:** Phyllis Messalina Gilch

**Affiliations:** Faculty of Law and Economics, University of Bayreuth, Bayreuth, Germany

**Keywords:** employer attractiveness, employer image, occupation, occupational fit, person-organization fit, recruitment

## Abstract

Organizations may need to attract occupational groups they did not recruit so far to implement strategic changes (e.g., digital transformation). Against the backdrop of this practical problem, this study introduces and explores an occupation-based measure of person-organization fit: occupational fit. I investigate its relationship with employer attractiveness based on human capital theory and explore the role of employer image as a moderator in this relationship. I surveyed 153 software engineers and mechanical engineers to analyze whether their occupational fit with software engineering and mechanical engineering firms is related to employer attractiveness. I find that occupational fit is only related to a firm’s employer attractiveness among software engineers. Employer image does not moderate this relationship. A qualitative follow-up study proposes first explanations for the unexpected differences between the two occupations by indicating that occupations may differ in the logic they apply to determine fit and their degree of professionalization. The study contributes to research by highlighting the neglected role of occupation in recruitment research and exploring potential boundary conditions of recruitment for fit. Implications for future research and practice are discussed.

## Introduction

During the digital transformation, software development has metamorphosed from a purchased service product into a vital core competency for many firms. This forces many organizations to adapt their workforce. Volkswagen, for instance, announced to create 2,000 additional jobs in the fields of software technology and electronic architecture (Paroway, 2020). However, creating jobs and filling the created vacancies are two different things. Like many other German technology firms, Volkswagen is a *pre-digital* company, i.e., it had been established before the digital age but now strives to hire employees in *digital* occupations. Hiring employees in digital occupations entails a matching problem on the labor market: The targeted job seekers do not perceive Volkswagen as a potential employer since they primarily associate the firm with mechanics and hardware production instead of software development. This picture of a pre-digital company does not fit with their digital occupation. This example leads to the question: Which role does an ‘occupational’ fit between job seeker and potential employer play in recruitment?

Job seekers’ occupation belongs to the less prominent characteristics studied in the literature on fit in recruitment, although occupations undeniably are closely linked to the working context and hence to employment decisions. We know that an occupational group commonly has a shared set of knowledge, skills, and abilities (KSAs) and values and a shared identity (Dierdorff and Morgeson, 2007; Skorikov and Vondracek, 2011; Dengler et al., 2016). Researchers frequently use these three categories of characteristics—KSAs, values, and identity—separately as content dimensions to operationalize fit (e.g., value-based person-organization fit) in the recruitment context (Kristof-Brown and Guay, 2011). Interestingly, although occupation conceptually comprises these dimensions, I found no study that has considered occupation as a content dimension of person-organization fit. Research has hardly paid attention to practice-oriented questions such as how to attract specific types of employees. It is thus unknown whether and how job seekers’ individual differences influence employer attractiveness in occupational training or education, and we lack comparisons between different groups of job seekers (Breaugh, 2013; Evertz and Süß, 2017). We thus lack research on occupations in recruitment and, more specifically, on ‘occupational’ fit.

While we lack knowledge on occupations in recruitment, we know much about fit measures. Many researchers have researched different fit measures, some closely related to ‘occupational’ fit (Kristof-Brown et al., 2005). On the one hand, research on person-vocation fit scrutinizes the match between persons’ interests and their occupational career choice based on vocational theories. On the other hand, investigations on person-job fit assess the match between persons’ KSAs and the jobs and tasks they (expect to) perform at work. However, to my knowledge, no investigation has tackled the problem of *Volkswagen* mentioned above and scrutinized the (non-)fit between the occupation of potential employees and the ‘occupation’ job seekers associate with an organization. Therefore, I try to fill this gap by studying the fit between job seekers’ occupation (reflecting their KSAs, values, and shared identity) and the ‘occupation’ of an organization, i.e., the tasks and jobs they expect to perform within the organization. Hence, I combine elements of person-vocation fit and person-job fit to create ‘occupational fit’ as a new measure of person-organization fit.

We do not know whether occupational (non-)fit—i.e., a match (or mismatch) between job seekers’ occupation and the occupation job seekers associate with the firm—substantially affects job seekers’ evaluation of employer attractiveness. Also, we do not know whether every occupational group includes occupational fit in their employment decision and, more generally, whether an occupation is a valid criterion to distinguish between recruitment target groups. We also do not know whether—like in the example of Volkswagen—firms’ efforts to recruit strategically relevant yet newly targeted occupational groups might be compromised by occupational non-fit and, if so, what employers could do to mitigate adverse effects. This lack of knowledge creates a blind spot in explaining job seekers’ employment decisions and ignores the potential additional explanatory value of occupation as a dimension of person-organization fit. Furthermore, it impedes our ability to validly anticipate the consequences of strategic recruitment decisions, such as a shift in target groups.

To fill this gap, this study introduces the concept ‘occupational fit’ as an interaction between a person’s occupation and the corresponding characteristic of the firm, i.e., ‘firm occupation.’ In a very early phase of their employment search, when no previous employment-related interaction between job seeker and organization had occurred, firm occupation reflects job seekers’ expectations of the tasks and jobs performed within the organization. This leads to the association of specific occupational group(s) performing these tasks and jobs. For example, Google, a company primarily known for its sophisticated and helpful search algorithms used in daily life, is likely to be associated with the software engineering occupation because employees from this occupation have the necessary skills to perform the tasks necessary to develop such algorithms. Thus, when software engineers are asked to rate Google as a potential employer, they may perceive themselves as a good “fit” to the company. In contrast, job seekers from a different occupation (e.g., mechanical engineering) may perceive a non-fit, since they do not envision themselves capable of performing those tasks. Whether and to what extent job seekers base their evaluation of employer attractiveness on ‘occupational fit’ is so far unclear. This study aims to fill this gap by examining the relationship between ‘occupational fit’ and attractiveness.

To embed the investigation on occupational fit in existing knowledge about the evaluation of attractiveness, I introduce employer image as moderating variable. Besides perceptions of fit, image is one of the most critical factors influencing employer attractiveness (Uggerslev et al., 2012). In the case of job seekers depreciating occupational non-fit, employers high in image ratings may be curious whether they can nevertheless rely on the positive impact of their image. Based on the above example, I assume that software engineers might be less inclined to apply for a job at the ‘mechanical’ Volkswagen company caused by their occupational non-fit. Volkswagen, however, may want to know whether it can overcome this hurdle by capitalizing on its strong positive employer image. Hence, I will additionally study employer image as a variable potentially interacting with occupational fit.

Based on data from two quantitative online surveys (pre-study and main study) among German software engineers and mechanical engineers and a qualitative post-study, this study contributes to existing knowledge in two ways. First, it extends the literature on person-organization fit by proposing occupation as an additional content dimension for fit assessment in the decisive early phase of job search. Occupation of both job seeker and organization is an easily accessible characteristic (Miller, 2013), making it a valuable and straightforward tool for fit assessment, although job seekers may only have limited information about the employer. This distinguishes the content dimension occupation from hitherto known dimensions of person-organization fit, like values or identity, since their assessment requires a more profound examination of a potential employer. Also, I extend the range of job seekers’ individual differences relevant for job search by focusing this study on the yet neglected criterion ‘occupation.’ The study thereby helps to solve the puzzle on what information job seekers use to make inferences about employers in the early stage of recruitment, as Breaugh (2013) requested in his review on employee recruitment. Second, this study creates awareness for potential boundary conditions of recruitment for fit. On the one hand, it discovers that it is yet unclear whether employer image amplifies or weakens the positive (negative) effect of fit (non-fit). However, since image and person-organization fit belong to the most important antecedents of employer attractiveness (Uggerslev et al., 2012), future research needs to determine which circumstances guarantee a positive effect of image. On the other hand, this article points to the challenges of the flip side of recruiting for fit, which is ‘non-fit’ recruitment. A large body of research investigates the nature and consequences of recruitment for person-organization fit (Kristof-Brown and Guay, 2011). However, it neglects that employers may need to address non-fit target groups specifically, frequently for strategic reasons. This, of course, has different implications than recruitment for fit and challenges the predominant paradigm of fit-oriented recruitment.

## Theoretical background and hypotheses

### Employer attractiveness and fit theory

In times of strategic changes, recruitment holds the challenging task to ensure the organization’s supply with employees who possess the KSAs to enact its new strategy (Gilch and Sieweke, 2021), which may afford to recruit a new occupational target group. Attractiveness, a central determinant of recruitment outcomes, deserves specific attention in this context. Attractiveness signifies “the degree in which a person favorably perceives an organization as a place to work” (Rynes, 1991). It reflects job seekers’ attitudes towards employers. Previous research has shown that attractiveness precedes many variables that determine recruitment success (Chapman et al., 2005; Uggerslev et al., 2012). For example, attractiveness directly affects job seekers’ intention to apply (Thoms et al., 2004) and their intention to pursue the application process (Lee et al., 2013). Attractiveness indirectly benefits the employer by enhancing recommendation intention (Ritz and Waldner, 2011; Tsai et al., 2015), the accuracy of information recall, and job seekers’ motivation to engage with the company (Walker et al., 2012). Recruiters thus strive to provoke positive attractiveness evaluations among potential applicants.

One of the most important antecedents of attractiveness is person-organization fit (Uggerslev et al., 2012). Fit describes a state of alignment of an individual’s characteristics and organizational characteristics (Kristof-Brown et al., 2005). Person-environment fit is one of the most frequently applied theoretical perspectives to elucidate the relationship between job seekers’ characteristics and an employer (van Vianen, 2018). The roots of the notion of fit lie in interactional psychology, where positive outcomes result from the interaction between person and environment (Kristof-Brown and Guay, 2011). The most fundamental principles underlying fit theory are that (a) outcomes can more reliably be predicted by fit than by the single components of fit and (b) a match between personal and environmental characteristics provokes the most favorable outcomes (van Vianen, 2018). This matching of personal and environmental characteristics has been subject to numerous studies in the recruitment context where job seekers compare their characteristics with characteristics of potential employers to determine whether fit is present or not. In this context, we know various types of person-environment fit, with person-vocation fit, person-job fit, and person-organization fit being the most prominent examples.

Individuals base their evaluation of fit on several content dimensions. They compare their characteristics with the characteristics they attribute to the employer within a specific dimension, determining fit or non-fit (Kristof-Brown et al., 2005). Among the dimensions frequently applied to various types of fit are preferences, needs, personality, identity, values, and KSAs (Kristof-Brown et al., 2005; Su et al., 2015). Generally, fit can be operationalized on any range of personal characteristics and organizational characteristics (Harrison, 2007; Kristof-Brown and Guay, 2011). In the recruitment context, content dimensions commonly relate to the work environment (Kristof-Brown et al., 2005; Kristof-Brown and Guay, 2011). An example of this is the value-based measurement of person-organization fit founded on the influence of values on job seekers’ attitudes and their prospective colleagues’ attitudes.

### Occupation as a new content dimension of fit

Occupation, the subject of this study, has not served as a content dimension of fit so far. However, it has the power to differentiate individuals along three established content dimensions (KSAs, values, and identity) and substantially relates to the work environment. First, occupational groups differ from each other by their KSAs, since an occupation includes “both technical or procedural expertise and the normative and prescriptive principles” (Alutto et al., 1971) and enables individuals to execute “jobs that share extensive commonalities in their required skills and tasks” (Dengler et al., 2016). Second, occupational groups gather individuals with similar values. Because individuals attempt to match their occupation with their self-concept, they choose occupations that match their values (Super, 1953; Kristof, 1996; Holland, 1997; Su et al., 2015). Studies on person-vocation fit and vocational choice have proved this relationship (Kristof, 1996; Elias et al., 2018). Third, occupations differentiate between job seekers according to their social identity since individuals acquire an occupational identity through formal education, task-specific learning by doing, and exposure to the occupational peer group (Mason and Mudrack, 1996; Gibbons and Waldman, 2004; Miller, 2013). Acting and learning in their occupational social context provide them with occupation-specific experiences and social resources such as information, influence, and status over time that shape their identity (Lin, 2001). Occupation thus is a composite of three established content dimensions of person-organization fit (KSAs, values, and identity). Beyond that, occupation comprises the interactions and intersections between its composites and thereby reflects the unique experiences that shape individuals’ perceptions of a particular occupation. Moreover, occupation is by nature closely linked to the work environment of individuals and hence fulfills the criteria of a content dimension of person-organization fit.

Applying the content dimension ‘occupation’ to person-organization fit yields a measure of fit where job seekers compare their occupation to the occupation they associate with a potential employer, i.e., the jobs and tasks they expect to perform when working with a potential employer. I will name this type of fit ‘occupational fit’ in the following. Occupational fit allows job seekers to evaluate fit based on occupation as a ‘superficial’ criterion instead of comparing their ‘deep-level’ characteristics, such as values or social identity, to corresponding employer characteristics. Deep-level characteristics may be hard to assess for job seekers in the early phase of their job search since they only possess limited knowledge about employers (Breaugh, 2013). In proposing occupational fit, I assume that job seekers heuristically assess information on employers by focusing on a content dimension that is easily accessible to them due to its close relatedness to the working and job searching context (Miller, 2013).

### The relationship between occupational fit and employer attractiveness

According to fit theory, it is rewarding and thus desirable if a person and its environment match (Kristof, 1996; Kristof-Brown and Guay, 2011). The similarity-attraction paradigm explains this mechanism by suggesting that individuals appreciate an environment similar to their selves and that this similarity predicts attraction (Byrne, 1971, 1997) because “individuals favor stimuli that reinforce the logic and consistency of their world” (Montoya et al., 2008). Thus, the more job seekers perceive an organization to be similar to their characteristics, the more attractive they perceive the organization as a potential employer.

Beyond this general reasoning applicable to several fit dimensions, human capital theory explains the relationship between fit and attractiveness regarding the occupational dimension. This complementary explanation is somewhat tangible: Occupational similarity promises to compensate for educational or training expenses and higher wages. Individuals usually choose to train for an occupation to enhance their human capital in income-generating abilities, i.e., occupation-specific KSAs they need to exercise an occupation to earn their living. Acquiring human capital implies a certain period of education, training, and learning, which may come at the cost of educational/training expenses (Becker, 1994). Individuals aim to be reimbursed for these expenses by finding a job that best covers their expenses by being well-paid. Wage, in turn, increases with a worker’s level of occupation-specific experience as a form of human capital (Kambourov and Manovskii, 2009; McDonald, 2011). The level of occupational experience rises with the time working in a particular occupation by a further accumulation of occupation-specific KSAs, which enhance working productivity (Becker, 1994). Since occupational experience goes along with acting and learning in social contexts, individuals gain occupation-specific social resources such as information, influence, and status over time (Lin, 2001). By increasing a worker’s human capital, occupational experience implies positive outcomes for the individual, e.g., in terms of better chances for promotion to higher job ranks (Kwon and Meyersson Milgrom, 2014) or higher wages (Kambourov and Manovskii, 2009; Nordin et al., 2010; Sullivan, 2010). Hence, occupational experience increases individuals’ ability to compensate for their investment in education and training by gaining higher wages. Therefore, job seekers are attracted to employers that offer the chance to gain as much occupation-specific experience as possible. Job seekers can best achieve this by choosing an employer that provides occupational fit since occupational fit signals the promise to gain experience in the typical jobs and tasks of the job seekers’ inherent occupation. I thus hypothesize:

*Hypothesis 1*: Occupational fit is positively related to employer attractiveness.

### The moderating effect of employer image

Although person-organization fit is a strong predictor of attractiveness, the predictive value of the employer image in the recruitment context may not be underestimated (Uggerslev et al., 2012). Image “reflects an amalgamation of mental representations and associations regarding an organization as an employer” (Lievens and Slaughter, 2016) and is fed by associations with the employer brand as well as with information generally linked to the employer, such as product or media experience (Kim et al., 2011). Previous findings suggest that the most critical image facets such as high income, attractive location, or good work-life balance are tangible attributes associated with an organization and reflect job seekers’ perception of the utilitarian value of an employer (Lievens and Highhouse, 2003; Tsai and Yang, 2010; Baum and Kabst, 2013; van Hoye et al., 2013; Yu, 2014). Thus, the image score shows how strongly job seekers assume that the company meets their needs and makes them feel comfortable. This comfortableness triggers loyalty with an employer during employment and prevents employees from leaving (Ito et al., 2013; Alshathry et al., 2017). A positive image thus has the power to extend job seekers’ period of employment. On the downside, negative image signals low utilitarian value to job seekers and thereby reduces their expected duration of stay.

Combined with the above reasoning grounded in human capital theory, image may alter the relationship between occupational fit and attractiveness. In a scenario of occupational fit, job seekers see the chance to increase their occupational experience and thereby gain higher wages to compensate for their investment in education and training. Compared to a negative-image employer, job seekers expect a more extended stay at a positive-image employer: A positive image indicates that they feel the employer can fulfill their values and needs. Since this is a goal of the search for an employer, this fulfillment of values and needs, once obtained, reduces the urgency to continue the search for alternative employers and thereby extends employment tenure. An extended stay in an occupationally fitting surrounding increases their expected gain in occupational experience. Hence, it increases their ability to compensate the investment in their human capital to a more considerable degree than at a negative-image employer. Job seekers thus may find positive-image employers more valuable in this regard and hence more attractive. Positive-image employers thus benefit from occupational fit to a larger extent than negative-image employers. I thus hypothesize:

*Hypothesis 2*: Employer image amplifies the positive relationship of occupational fit and employer attractiveness, such that the relationship is stronger if employer image is positive.

## Materials and methods

### Operationalization of occupational fit

This study measures occupational fit objectively and indirectly, which means that I measure job seekers’ and organizational characteristics separately instead of directly asking job seekers about their fit perception. For this methodology, it is essential to compare the person’s and the organization’s characteristics along a commensurate content dimension, meaning that job seekers, for example, compare their values to the employer’s values or their personality to the personality traits associated with the employer (Kristof-Brown et al., 2005). Hence, I operationalize occupational fit as an interaction between job seekers’ occupation and ‘firm occupation’ since occupational fit displays an occupation-based match between job seekers’ and organizations’ characteristics.

While job seekers’ occupations can be assessed straightforwardly, the ‘firm occupation’ assessment deserves special attention. Firm occupation develops by job seekers making inferences on the ‘occupation’ of potential employers by collecting various associations with an organization. From early childhood on, individuals implicitly learn about their environment by perceiving information (Koch and Stahl, 2017), attaching it to their mental representations (i.e., brands) of objects around them (McAlister and Cornwell, 2010), and storing it as brand knowledge (Cable and Turban, 2001). Researchers found various information transferred to employer brands, e.g., personality traits and demographic characteristics such as gender, age, or class (Levy, 1959; Aaker, 1997; Grohmann, 2009; Hohenberger and Grohs, 2020). In a similar vein, job seekers attribute a certain ‘firm occupation’ to an employer: From personal experience with a firm, its products or services, media coverage, or word-of-mouth, job seekers know which tasks and jobs are performed within an organization and can thus form an expectation about which occupational group works for an employer or has a good chance of being hired. Additionally, active job seekers perceive which occupation an employer is predominantly hiring with the aid of job advertisements. Thus, the ‘firm occupation’ generated from these inferences reflects the impression about which occupational group an organization mainly employs and depicts the organization-related component of occupational fit.

Since I chose to focus this investigation on mechanical and software engineering occupations, I need to identify firms with firm occupations that either fit or do not fit with these two occupations. Hence, I conduct a pre-study to identify ‘mechanical engineering firms’ and ‘software engineering firms’, which serve as stimuli for the main study.

### Pre-study

#### Sample and procedure

Because I aimed to include four companies’ logos as stimuli in the main study (two mechanical engineering firms, two software engineering firms), I conducted a pre-study to identify firms with respective firm occupations. The participants of the pre-study each rated three out of twelve^1^ firms, whereby each firm’s logo served as a stimulus. The sample of 12 firms was selected considering two criteria: First, organizations had to be ranked in well-known German employer rankings such as the German Universum ranking and ‘arbeitgeber-ranking.de’ to ensure a certain degree of prominence within the target population. Second, organizations had to belong to either the mechanical engineering sector or the software engineering sector to increase the probability of finding firms with a clear mechanical or software engineering firm occupation.

As I defined firm occupation as the “occupation mainly employed by the organization,” respondents rated firm occupation by completing the sentence “This company mainly employs….” The three answers provided were “… mechanical engineers,” “… software engineers,” and “… other occupations,” and all had to be rated on a scale from 1 (“I strongly disagree”) to 7 (“I fully agree”).

I conducted an online-based survey among persons working for a large multi-national technology company and its filial companies distributed across several business locations within Germany. I provided supervisors of all levels of hierarchy from various mechanical and software engineering departments with invitation letters they could use to invite their employees to participate in this study. The invitation letter additionally asked participants to spread the survey link among their colleagues to increase the number of participants. In total, 223 respondents completed the pre-study. Of those, I excluded 64 because they neither had an occupational background in mechanical engineering nor software engineering or failed to provide this information. Of the remaining 159, the majority had an occupational background in mechanical engineering (81.1 percent). Typical for the occupations in question (Statista Research Department, 2021), the majority were male (86.2 percent). Respondents’ ages ranged from 18 to 60 years, and 54.1 percent were between 21 and 30.

#### Results

I calculated a one-factorial ANOVA to determine which two firms from each category of firm occupation had the clearest firm occupation to best serve as stimuli for the main study. I conducted the ANOVA with repeated measurements for each company to see whether all three occupational groups (mechanical engineers, software engineers, other occupations) were perceived to be equally present or whether there is a dominant occupational group in the respective company. The results (*cf.* Table 1) show that the strength of the three occupational groups differs significantly in all companies. The firms 3, 6, 8, 10, and 12 are dominantly associated with the occupation ‘mechanical engineering’ and the firms 2, 7, 9, and 11 with ‘software engineering.’ Firm 1 dominantly employs ‘other occupations’ and is not suitable for the main study. Contrasting each firm’s mean score in ‘mechanical engineering’ against ‘software engineering,’ a comparison of absolute mean differences signals that firms 8 and 12 had the clearest mechanical engineering firm occupation and firms 7 and 11 had the clearest software engineering firm occupation. Thus, I chose these two pairs of firms as stimuli for the main study.

**Table 1:** Results of firm occupation analysis (pre-study).

		**“Mainly employed occupation”**						**ANOVA (repeated)** ^*^			**Contrast mechanical *vs*. software engineers**
		**Mechanical engineers**		**Software engineers**		**Other** **occupations**					
**Firm**	**N**	**Mean**	**SD**	**Mean**	**SD**	**Mean**	**SD**	**df**	**F**	**p**	Absolute mean difference^**^
**Dominant occupation: mechanical engineering**											
8	44	5.27	1.66	2.55	1.27	4.48	1.52	(2, 86)	42.08	<0.001	**2.73**
12	41	5.85	1.17	3.78	1.29	4.66	1.17	(2, 80)	44.47	<0.001	**2.07**
10	38	6.13	0.99	4.63	1.63	4.74	1.55	(2, 74)	17.81	<0.001	1.50
3	32	6.06	1.13	5.34	1.43	4.91	1.33	(2, 62)	9.54	<0.001	0.72
6	36	5.50	1.36	4.83	1.68	4.42	1.57	(2, 70)	6.35	<0.01	0.67
**Dominant occupation: software engineering**											
11	44	2.61	1.30	6.50	0.85	4.20	1.42	(2, 86)	109.92	<0.001	**3.89**
7	57	3.32	1.64	6.75	0.58	4.54	1.45	(2, 112)	118.31	<0.001	**3.44**
2	40	3.58	1.52	6.38	0.70	4.13	1.36	(2, 78)	65.65	<0.001	2.80
9	35	3.94	1.41	6.00	1.26	3.80	1.21	(2, 68)	31.84	<0.001	2.06
**Dominant occupation: other occupations**											
1	40	2.28	1.13	4.90	1.32	5.63	1.35	(2, 78)	67.86	<0.001	2.63

* The given value of p includes a Huynh–Feldt correction for lack of sphericity.

** The two firms highest in ‘absolute mean difference’ in the categories mechanical engineering and software engineering are printed in bold and used as stimuli for the main study.

### Main study

#### Sample and procedure

I surveyed potential applicants (students, graduates, professionals) with either mechanical engineering or software engineering backgrounds in Germany *via* an online questionnaire to test my hypotheses. I spread the link to the questionnaire together with a short note on the survey’s purpose using the social networks Facebook, LinkedIn, and Xing, which the target population strongly frequents. Additionally, I asked participants to spread the survey link among their peers to reach more potential respondents. Participants received the offer to participate in a lottery after completion, in which they could win no-cash prizes amounting to 25 euros.

Of the 481 persons who started the survey, 273 (56,76 percent) completed it.^2^ I excluded 120 responses from the sample due to missing values (e.g., occupation not indicated) or because they did not fit the targeted population (occupational background, e.g., in social sciences). The final sample consisted of 153 participants (81.7 percent male) and contained only complete datasets. The respondents’ age ranged from 18 to 60 years, of which 69.3 percent lay between 21 and 30 years. The majority (56.9 percent) had between one and 5 years of work experience. Despite intense efforts to equally address both occupational groups, the respondents with a background in mechanical engineering (72.6 percent) outnumbered the software engineers.

I opted for a between-subjects design to minimize carry-over effects and the risk of dropouts. I programmed the survey software tool to randomly assign each respondent to one of the four organizations identified in the pre-study. Respondents thus had a chance of 50 percent (2 out of 4) to fall into either the fit or the non-fit condition. For example, a software engineer was randomly selected to evaluate one out of the four firms and thereby had a chance of 50 percent to be drawn a software engineering firm. While I expected each firm to be rated by 25 percent of all cases, actual shares ranged from 22.9 percent (35 of 153) to 26.8 percent (41 of 153). The unequal distribution of excluded cases causes deviations from perfect randomization. As stimuli, I used the official logos of the four employers.

#### Measures

*Dependent variable:* I used the attractiveness scale on the general attractiveness of an organization (Highhouse et al., 2003), shortened to three items by Baum and Kabst (2014). A sample item was “For me, this company would be a good place to work.” Respondents evaluated these items on a 7-point Likert-type scale ranging from 1 (“I strongly disagree”) to 7 (“I fully agree”). The reliability of the scale was very good (α = 0.94).

*Independent variables:* Consistent with related studies of fit measures and suggestions of interactional theory, the fit measure ‘*occupational fit*’ is depicted by an interaction term between the two independent variables ‘firm occupation’ (derived from the pre-study) and ‘job seeker occupation’ (Edwards, 1994; Ehrhart and Ziegert, 2005; Schreurs et al., 2009). I coded firm occupation “0” for software engineering firms and “1” for mechanical engineering firms in the main study. The second component of occupational fit, *job seeker occupation*, was assessed by asking, “Which occupational background do you associate with (for example, through education, studies or work experience)?” and takes the value “0” for mechanical engineering and “1” for software engineering. Occupational fit is present when a mechanical engineer evaluates a mechanical engineering firm (n = 58) and when a software engineer evaluates a software engineering firm (n = 24). The remaining two combinations, mechanical engineers evaluating software engineering firms (n = 53) and software engineers evaluating mechanical engineering firms (n = 18), belong to the non-fit condition.

*Moderator:* Employer image was measured using the eight-item scale from Collins (2007) and Collins and Stevens (2002) in the form used by Baum and Kabst (2013). It comprises items such as above-average income, attractive working locations, or good opportunities for advancement. I excluded the item “This company offers exactly the job I want” ex-post since it semantically relates too closely to attractiveness. Item deletion increased reliability from α = 0.903 to α = 0.908.

*Control variables:* I checked for the potential confounding effects of several covariates. Unless stated otherwise, all items were measured on a 7-point Likert-type scale ranging from 1 (“I strongly disagree”) to 7 (“I fully agree”). First, I included employer familiarity and reputation due to their effect on attractiveness (Cable and Turban, 2001; Yu and Davis, 2019). Employer familiarity is “the level of awareness that a job seeker has of an organization” (Cable and Turban, 2001). I used a brand familiarity scale of product brand research origin from Delgado-Ballester et al. (2012) and adapted it to the recruitment context (Lievens et al., 2005). The three scale items (α = 0.87) are “This company is familiar to me,” “I have heard something about this company,” and “I know this company.” Employer reputation, which is defined as “a job seeker’s beliefs about the public’s affective evaluation of the organization” (Cable and Turban, 2001), was measured by a four-item scale (α = 0.94) from school context (Collins, 2007; Baum and Kabst, 2014), which I adapted to the working context. A sample item was “My friends have high regard for this company as an employer.” Third, I controlled for age, gender, and work experience (all dummy-coded). To provide their age, participants assigned themselves to one out of five age categories: less than 20 years old; 21–30 years old; 31–40; 41–50; 51–60. For gender, women were coded “0” and men “1.” Work experience was measured in the three categories “less than one year,” “1–5 years,” and “more than five years.” Potential company-specific effects are captured by including three firm dummy variables.

#### Analysis

I inspected the data for potential bias from common method variance to ensure data quality. Common method variance was accounted for both procedurally and statistically as proposed by Podsakoff et al. (2003). Data was collected from different sources to avoid common source effects: Pre-study participants provided information about firm occupation, whereas main study participants provided data for the other independent variables and attractiveness as dependent variable (Favero and Bullock, 2015). Statistically, common method variance is unlikely to be problematic in this analysis since the independent variables ‘job seeker occupation,’ and ‘firm occupation’ are manifest variables. Concerning the moderator ‘employer image,’ a latent variable, Siemsen et al. (2010) found interactions not to suffer from common method variance. A confirmatory factor analysis (CFA) with the marker variable technique^3^ (marker variable: creative efficacy) confirmed that the relationships between the substantive variables are not skewed (Williams et al., 2010; Simmering et al., 2015).

The subsequent test of hypotheses through a linear regression analysis was conducted in Stata 15. For this analysis, I centered all continuous predictors to their mean to increase the interpretability of results (Dalal and Zickar, 2012) and entered the variables into the regression line in three subsequent steps: First, control variables were included (Model 1). Second, I added the direct effects of the two components of occupational fit—job seeker occupation and firm occupation, and the two-way interaction between job seeker’s occupation and firm occupation, which depicts occupational fit (Model 2). Third, I included the direct effect of image and the two- and three-way interaction terms to depict the interaction between image and the components of occupational fit (Model 3).

## Results

### Main study results

Descriptive statistics, intercorrelations, value of ps, and scale reliabilities for latent constructs are depicted in Table 2. The high correlation between employer image and employer reputation (*r* = 0.74) demanded a test to see if the data met the assumption of collinearity. Results indicated that multicollinearity was not a concern (employer image: Tolerance = 0.31, VIF = 3.27; employer reputation: Tolerance = 0.41, VIF = 2.45).

**Table 2:** Variable description, correlations, and scale reliabilities.

**Variable**	**Mean**	**SD**	**1**	**2**	**3**	**4**	**5**	**6**	**7**	**8**	**9**	**10**
1. Age	–	–	–									
2. Gender *(1 = male)*	0.82	0.39	0.17^*^	–								
3. Work experience	–	–	0.69^***^	0.14	–							
4. Company	−	−	0.05	0.15	0.08	−						
5. Employer familiarity	5.03	1.74	0.09	−0.07	0.19^*^	−0.08	[0.87]					
6. Employer reputation	4.39	1.34	−0.11	−0.04	−0.04	0.05	0.41^***^	[0.94]				
7. Job seeker occupation *(1 = software engineering)*	0.27	0.45	−0.13	0.06	−0.01	−0.08	0.05	0.00	−			
8. Firm occupation *(1 = mechanical engineering)*	0.50	0.50	−0.03	0.03	−0.10	0.48^***^	−0.43^***^	−0.25^**^	−0.08	−		
9. Employer image	4.63	1.18	−0.15	−0.02	−0.02	−0.05	0.46^***^	0.74^***^	−0.00	−0.34^***^	[0.91]	
10. Employer attractiveness	4.09	1.52	−0.09	0.00	−0.05	0.06	0.25^**^	0.63^***^	0.06	−0.20^*^	0.60^***^	[0.94]

* *p* < 0.05;

** *p* < 0.01;

*** *p* < 0.001.

Cronbach’s alpha in brackets on diagonal.

**Figure 1:**
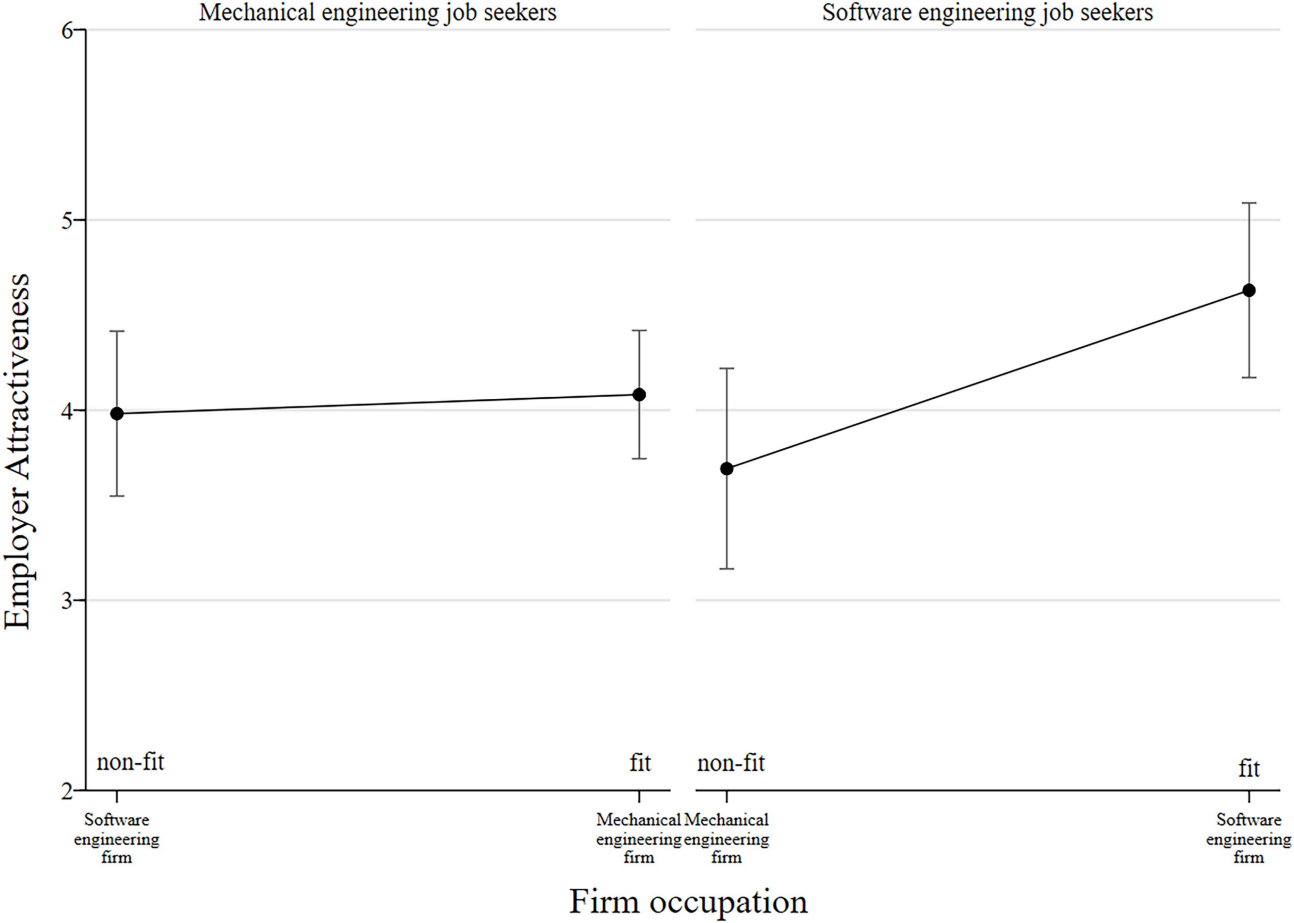
Interaction between firm occupation and job seeker occupation with 95% confidence intervals (Model 2).

The regression analysis results in Table 3 provide mixed evidence for the hypotheses. Concerning *hypothesis 1*, the interaction term between job seekers’ occupation and firm occupation in Model 2 is negative and significant (*b* = −1.04; *p* = 0.01). As the interaction plots illustrate (Figure 1), the data show differences between the occupational groups: Whereas occupational fit does not provoke significantly higher attractiveness ratings among mechanical engineers, it does so among software engineers. A contrast analysis shows that mechanical engineers value fit only with an insignificant increase in attractiveness of 0.10 compared to non-fit (95% CI: [−0.518; 0.718]). However, the difference between fit and non-fit among software engineering job seekers is significant, with a difference in attractiveness of −0.94 (95% CI: [−1.671; −0.204]). Therefore, the data partially support *hypothesis 1*.

**Table 3:** Results of linear regression analyses on employer attractiveness.

**Variables**	**Model 1**		**Model 2**		**Model 3**	
	b	(*value of p*)	b	(*value of p*)	b	(*value of p*)
**Age**						
Age = 21–30 years	−0.45	(0.21)	−0.35	(0.30)	−0.34	(0.32)
Age = 31–40 years	−1.09	(0.02)	−1.07	(0.02)	−0.88	(0.07)
Age = 41–50 years	−0.79	(0.20)	−0.79	(0.19)	−0.42	(0.52)
Age = 51–60 years	−0.09	(0.91)	−0.09	(0.90)	0.12	(0.86)
Gender	0.20	(0.42)	0.20	(0.41)	0.13	(0.62)
**Work experience**						
Work experience = 1–5 years	0.14	(0.57)	0.18	(0.50)	0.08	(0.76)
Work experience >5 years	0.32	(0.47)	0.42	(0.34)	0.26	(0.59)
**Company**						
Company = Firm 2	−0.67	(0.05)	−0.61	(0.03)	−0.55	(0.05)
Company = Firm 3	−0.15	(0.65)	−0.20	(0.52)	−0.13	(0.69)
Company = Firm 4	−0.13	(0.65)	0.00	(.)	0.00	(.)
Employer familiarity	−0.10	(0.21)	−0.15	(0.07)	−0.17	(0.03)
Employer reputation	0.70	(0.00)	0.70	(0.00)	0.49	(0.00)
Job seeker occupation			0.65	(0.03)	0.75	(0.03)
Firm occupation			0.10	(0.75)	0.29	(0.34)
Job seeker occupation X Firm occupation			−1.04	(0.01)	−1.30	(0.00)
Employer image					0.42	(0.02)
Job seeker occupation X Employer image					−0.09	(0.74)
Firm occupation X Employer image					0.03	(0.88)
Job seeker occupation X Firm occupation X Employer image					−0.25	(0.48)
N	153		153		153	
Adjusted *R*^2^	0.39		0.41		0.44	
F	11.93	(<0.001)	12.58	(<0.001)	13.87	(<0.001)

Gender: 0 = female; 1 = male; Company: 1&3: software engineering firms; 2&4: mechanical engineering firms; Job seeker occupation: 0 = mechanical engineering; 1 = software engineering; Firm occupation: 0 = software engineering; 1 = mechanical engineering.

**Figure 2:**
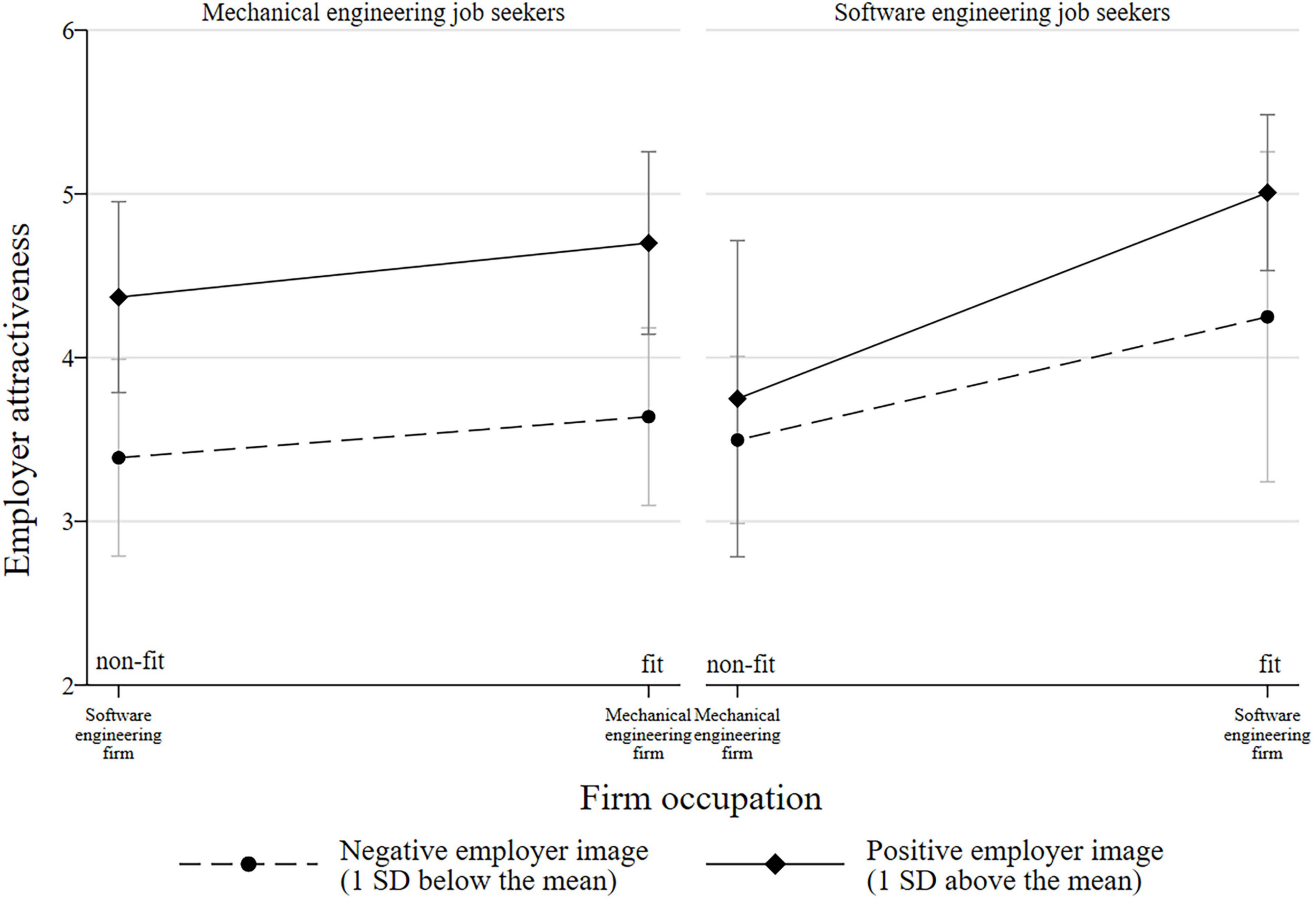
Interaction between employer image, firm occupation, and job seeker occupation with 95% confidence intervals (Model 3).

Model 3 presents the findings concerning *hypothesis 2*: Whereas the direct effect of image is significant (*b* = 0.42; *p* = 0.02), the three-way interaction term of job seekers’ occupation, firm occupation, and image does not prove significant (*b* = −0.25; *p* = 0.48). Figure 2 shows the interaction plot. Contrasting the results helps to understand the data better. Consistent with mechanical engineering job seekers not being sensitive to occupational fit in Model 2, the same pattern emerges in Model 3. Mechanical engineers do not differentiate significantly between fit and non-fit in both the negative and positive image condition (measured 1 SD below and above the mean, respectively): The difference in attractiveness between fit and non-fit amounts to 0.25 (95% CI: [−0.53; 1.03]) in the negative-image condition and 0.33 (95% CI: [−0.49; 1.15]) with a positive image. Meanwhile, mechanical engineers clearly differentiate by the level of image: The difference between negative and positive image alters the attractiveness rating significantly in case of fit (*Δ* = 1.06; 95% CI: [0.18; 1.95]) and non-fit (*Δ* = 0.98; 95% CI: [0.14; 1.82]). Mechanical engineers thus continue to show insensitivity towards occupational fit in the presence of image. The data on software engineering job seekers provides a different picture: Software engineers’ attractiveness rating does not differ between fit and non-fit in the negative-image condition (*Δ* = −0.75; 95% CI: [−1.90; 0.40]) but does so in the positive image condition (*Δ* = −1.26; 95% CI: [−2.22; −0.30]). Taking the opposite perspective by focusing on the effect of image again shows that software engineers’ attractiveness rating is not altered significantly by image, neither in case of fit (*Δ* = 0.76; 95% CI: [−0.29; 1.81]) nor of non-fit (*Δ* = 0.25; 95% CI: [−0.76; 1.27]). Thus, the software engineers’ insensitivity towards image, in general, contradicts their tendency to show sensitivity towards occupational fit in the case of a positive image. Hence, I reject *hypothesis 2*.

### Spotlight on the unexpected differences among occupational groups

The two observed occupational groups unexpectedly show differences in the relationship between occupational fit and employer attractiveness. To obtain first indications on the reasons behind this, I decided to dig deeper into the differences between the two occupations. Therefore, I conducted four problem-centered, semi-structured interviews with potential job seekers on their valuation of occupational fit (Witzel and Reiter, 2012). Of the interview partners (age: 28–41, working in Germany, all-male), two were mechanical and two software engineers. I conducted the interviews in German (3) or English (1) in August and September 2021, recorded *via* a video conferencing service (average duration: 20 min), and transcribed verbatim. In the analysis process, I first scoured the data for hints on explanations, then grouped them into broader categories of similar content, and afterward combined them with existing theoretical knowledge (Gioia et al., 2013). Notably, two themes emerged that potentially explain the differences between mechanical and software engineers: their logic behind the definition of occupational fit and their degree of professionalization.

Concerning the first theme, the interviews revealed that—depending on their occupation—job seekers base their definition of occupational fit on differing logics. When asked for the criteria they use to determine occupational fit, software engineers mentioned several specific criteria: a fit with their team’s educational background and mentality, or the end customer:

‘If the end customers are […] housewives, for example, that is not that interesting for me. […] For me, the most interesting problems are when the end-users are developers’.

In contrast, mechanical engineers aim to meet employers’ needs and hope that the company sees them adding value. Thus, while mechanical engineers ask, ‘Who wants me?’, software engineers instead seem to focus on the question ‘What do I want?’. This may be caused by the fact that software engineering currently is a shortage occupation that is highly sought after in the labor market due to digital transformation. Software engineers know their power to decide which employer to choose (Kerkmann, 2019; Gilch and Sieweke, 2021). Mechanical engineers, by contrast, do not face such excessive labor demand and may hence be less inclined to ‘choosy’ behavior.

Concerning the second theme, the degree of professionalization, the software engineering occupation meets the criteria of a profession to a more considerable degree than the mechanical engineering occupation. Professions in their pure form show three key features: sharing a particular knowledge base, regulating and controlling the knowledge base, and sharing a common ideology that defines appropriate behavior (von Nordenflycht, 2010). My interview partners from both occupations asserted to need the knowledge they acquired during their studies or training to exercise their occupation. Hence, both occupations meet the first criterion of a profession by relying on a particular knowledge base. However, they do not regulate or control the specific content of the knowledge base, e.g., issuing legal professional status certificates as typical in the law profession. Therefore, both occupations do not meet the second criterion. However, differences occur regarding the third criterion, ‘ideology.’ While mechanical engineers’ interview answers do not contain hints for a professional ideology, software engineers mentioned several related points. By stating,

‘I think what unites all people in the computer science field is how you were trained to think. (…) When you do that all day, it also changes the way you think about problems, the way you deal with other people,’

one interviewee describes that his professional training shapes his internalized preferences (Frederickson and Rooney, 1990; Leicht and Lyman, 2006). Likewise, software engineers seem to have a strong preference for autonomy, one of the central professional norms (Alvesson and Kärreman, 2006; von Nordenflycht, 2010):

‘If you see a problem as a computer scientist, you want to have the autonomy to say: Let’s tackle it! (…) It can be discussed, but how the status quo is often simply accepted in other fields is incredibly difficult for computer scientists.’

Beyond this, one interview partner utters a negative attitude towards non-professionals in leading positions which is another sign of professional ideology (von Nordenflycht, 2010):

‘If there [in management] are guys who don’t have any clue about the technical aspects, (…) this would be a red flag for me.’

Altogether, neither mechanical engineering nor software engineering fully meets the standards of a profession. However, software engineering shows a higher degree of professionalization and can thus be called a ‘semi-profession’ (Etzioni, 1969).

Both themes, logic behind occupational fit and degree of professionalization, support the main study’s finding that software engineers are inclined to value occupational fit stronger than mechanical engineers.

## Discussion

This article explores the challenge of recruiting atypical occupational groups by proposing occupation as a content dimension of person-organization fit, i.e., occupational fit, and scrutinizing its relationship with attractiveness. The survey results provide some first empirical evidence about the nature of this relationship. Occupational fit is positively related to attractiveness—at least among one of the two occupations, or to put it the other way round: Occupational non-fit, presumably present when recruiting atypical occupational groups, leads to less positive attractiveness ratings—but only among one of the two occupations. In combination with the finding that image does not alter this relationship, recruiting atypical occupational groups appears as a task that is not easy to accomplish in the short run. Occupational groups essential for the company’s future viability are hard to attract as long as occupational groups ‘typical’ for the company dominate a company’s firm occupation. In the following section, I discuss implications for research and practice and limitations and directions of future research.

### Implications for research

This study contributes to current knowledge in two ways. First, this study highlights the role of occupation in recruitment by finding that (a) occupation is a content dimension of fit and (b) that job seekers’ evaluation of employers changes with job seekers’ occupation. As mentioned above, prior research offered little knowledge on the role of occupation in recruitment. This study provides first indications of empirical evidence for occupations’ influence on attractiveness *via* its function as a content dimension of person-organization fit. Occupation complements the known range of content dimensions such as values, KSAs, identity, or personality by a work-related, easily accessible facet. Whereas other content dimensions afford job seekers to possess deep knowledge about the employer (e.g., about one’s own and an employer’s values or personality), occupational fit is an accessible surface-level characteristic that allows job seekers to evaluate fit quickly (Miller, 2013). Since limited, superficial information on employers characterizes the early phase of a job search, occupational fit may be especially relevant for job seekers in this impactful early stage of their job search (Turban, 2001; Breaugh, 2013).

Beyond an occupation’s role as a content dimension of fit, the main study’s findings supplemented by the post-study found that job seekers evaluate employers differently depending on their occupational group. This finding contributes to the literature on ‘individual differences’ in recruitment, which investigates how the perception of employer attractiveness varies with job seekers’ attributes (Breaugh, 2013; Evertz and Süß, 2017). Occupation has not yet found great attention as an individual difference. My results, however, provide first evidence that occupation is a relevant criterion to distinguish between groups of job seekers since job seekers’ occupation decides on whether they take occupational fit into account. Additionally, regarding the post-study, it may be rewarding to consider the degree of professionalization of job seekers’ occupations in individual differences in recruitment. If non-professionalized companies aim at recruiting employees from professionalized occupations, job seekers may disapprove of the difference in professionalization between their current and their new job situation. For example, it could be relatively easy for a manufacturing firm to poach incumbents of the low-professionalized occupation mechanical engineering from a mechanical engineering firm. At the same time, it may be harder for a manufacturing firm to attract lawyers from a law firm since they belong to a highly professionalized occupation and would have to leave their professionalized environment. Differentiating job seekers according to their occupation or its degree of professionalization hence allows recruiters to target recruitment measures to each group and enhance the effectiveness of recruitment efforts (Doverspike et al., 2000; Newman and Lyon, 2009; Breaugh, 2013).

Second, this study sensitizes for boundary conditions of recruitment for fit by (a) exploring the role of employer image for occupational fit and by (b) addressing recruitment challenges for non-fit. In the main study, I attempted to shed light on the interplay between fit and image. However, the data did not support the hypothesized interaction with occupational fit. Against the backdrop of the failure to show a direct effect of occupational fit on attractiveness among mechanical engineers, it was expectable to equally not find an interaction effect within this group. Conversely, however, I would have expected an interaction among software engineers, but data did not prove significance either. One way to explain this non-finding is to assume a compensatory effect of image. A job seeker’s positive image score could trigger the feeling of ‘wanting to belong’ to this employer. If this feeling was comparatively strong, such that image would outperform occupational fit, it could make the job seeker ignore part of the non-fit information (Überschaer et al., 2016). However, we lack research on the interplay between image and fit in general, which would clarify the relationship of these two critical antecedents of attractiveness (Uggerslev et al., 2012).

As another potential boundary condition, this study sensitizes for companies’ challenge to sometimes recruit atypical target groups (recruitment for non-fit) deliberately and voluntarily. The main study proposes a negative relationship between occupational non-fit and attractiveness. Difficulties may result if occupational groups are the target of a strategic recruitment campaign but do not perceive themselves as fitting to the recruiting company. Digital Transformation is a setting where organizations strive to recruit atypical target groups, but it is not the only one (Barrick and Parks-Leduc, 2019). Take the example of a long-established but financially stricken stationary trade company that decided to reevaluate its current business model during a phase of strategic transition. It might need employees with entrepreneurial skills in business model innovation to be able to refresh its traditional business model with new perspectives. Recruiting for non-fit is an unoccupied field of research with specific challenges such as sending (employer) and receiving (job seeker) mixed signals or handling cognitive dissonance (Festinger, 1957; Pernkopf et al., 2021). Whereas we are well-informed about the contingencies and outcomes of recruiting for fit, research is necessary on the question of how recruitment for non-fit can succeed despite the observed challenges.

### Implications for practice

This study’s findings offer implications on handling occupational (non-)fit for organizational practice. For organizations, it is viable to recognize that occupational non-fit is negatively related to attractiveness. Hence, organizations that strive to recruit a new occupational group may aim at finding out whether this group is sensitive for occupational fit and then consider re-targeting recruitment messages. These messages should be recognizably addressed towards the new group and be authentic. It seems inadvisable for a pre-digital company producing mechanical systems to present itself as a complete software engineering company if a short internet search would convince the job seeker of the opposite. Such organizations may want to carefully consider to which degree they want to present themselves as ‘occupationally fitting’ without losing credibility. For example, they could frankly address the organization’s shift in target groups, communicate why, and paint an authentic picture of the diverse occupational groups currently employed. In the same breath, the employer may want to signal how it provides job seekers with opportunities to gain occupation-specific experience and achieve competitive wages.

Such successful recruitment for atypical occupational groups requires strategic management to timely involve recruitment in their planning. Recruiting atypical occupational groups is not trivial and requires tact, finesse, and thorough preparation. As soon as management intends to modify its business model or processes and thereby implicates recruiting an atypical occupational group, it is advisable to instruct recruitment to accompany this change. Due to its function as a ‘sensory organ’ of the organization, the recruitment department unites experience and knowledge about specific occupational groups and knows how to acquire information about the new group (Gilch and Sieweke, 2021). Acting as a ‘sensory organ’ entails sensitivity in designing appropriate recruitment measures and awareness for the preferences of the target groups. Compared to an *ad hoc* instruction to hire an atypical group, diligent conceptualization, and design of recruitment measures may allow recruitment to prevent or at least attenuate the negative consequences of occupational non-fit.

### Limitations and future research

One might be concerned about sampling issues that may limit the validity of findings in the quantitative study. The low number of participants from the software engineering occupation skews the findings for this occupational group. Therefore, I am cautious about assuming the generalizability of the results. However, the post-study’s findings support the notion of substantial differences between the occupations—although a more extended qualitative investigation would potentially support the findings more fundamentally. Considering the restricted sample size in the quantitative part, the significant relationship between occupational fit and attractiveness among software engineers is even more substantial. Furthermore, the focus on an objective assessment of fit limits the study. Objective fit reflects the actual fit level, allows verifying similarity without necessarily being involved in the situation, and predicts subjectively perceived fit (Kristof, 1996; Dineen et al., 2002). Research suggests that subjective fit may outperform objective fit in its predictive value of recruitment outcomes and therefore proposes subjective fit as a mediator between objective fit and attractiveness (Judge and Cable, 1997; Verquer et al., 2003; Überschaer et al., 2016). However, objective fit is crucial in settings where no interaction between the subjects had occurred previously (Montoya et al., 2008). Hence, objective fit indeed was suitable for measuring fit here. Since I aimed to initially explore the potential relationship between occupational fit and attractiveness in a very early recruitment phase, I did not include subjective occupational fit. Future research may address this omission, e.g., by including subjective occupational fit in the research model or by inquiring about its formation. Future research in this field may also aim to empirically investigate the relationship between objective occupational fit and person-organization fit or person-job fit to prove the additional explanatory value of occupation as a content dimension of fit. Additionally, future research would benefit from knowledge on the assessment of occupational fit, e.g., by reusing the indirect assessment method used in this study and comparing it to alternative approaches such as direct measurement. The latter would require the development of a measurement scale for direct occupational fit assessment, which would simplify the assessment of job seekers’ respective expectations towards future employers. This comparative work would provide deeper insights into the nature and formation of occupational fit. Finally, another potential area of future research is the investigation of firm occupation. Researchers may study the temporal stability of firm occupation and the mechanisms behind job seekers’ perceptions. From employers’ perspective, knowing how to actively shape firm occupation to facilitate fit perceptions of desired target groups would be rewarding.

Overall, this study introduced an occupation-based measure of person-organization fit and investigated its relationship with attractiveness and employer image. Results showed a positive connection between occupational fit and attractiveness for one occupation. Image did not moderate this relationship. The study complements extant research by underlining the importance of occupation and by creating awareness for the challenge of recruiting ‘atypical’ employees.

## Data availability statement

The raw data supporting the conclusions of this article will be made available by the author, without undue reservation.

## Ethics statement

Ethical review and approval were not required for the study on human participants in accordance with the local legislation and institutional requirements. The participants provided their written informed consent to participate in this study.

## Author contributions

The author confirms being the sole contributor of this work and has approved it for publication.

## Funding

This work was funded by the Deutsche Forschungsgemeinschaft (DFG, German Research Foundation)—491183248, the Open Access Publishing Fund of the University of Bayreuth, and the University of Bayreuth Graduate School.

## Conflict of interest

The author declares that the research was conducted in the absence of any commercial or financial relationships that could be construed as a potential conflict of interest.

## Publisher’s note

All claims expressed in this article are solely those of the authors and do not necessarily represent those of their affiliated organizations, or those of the publisher, the editors and the reviewers. Any product that may be evaluated in this article, or claim that may be made by its manufacturer, is not guaranteed or endorsed by the publisher.

## Footnotes

^1^Firm 4 and Firm 5 had to be excluded from analysis because they employed the majority of respondents.

^2^Among the 481 were 110 persons (22.87 percent) who dropped out on the first two survey pages (14 pages in total). However, high rates of early dropout have been found to be common in online surveys (Hoerger, 2010).

^3^Results available upon request.

## References

[ref1] Aaker J. L. (1997). Dimensions of brand personality. J. Mark. Res. 34:347. doi: 10.2307/3151897

[ref2] Alshathry S. Clarke M. Goodman S. (2017). The role of employer brand equity in employee attraction and retention: a unified framework. Int. J. Organ. Anal. 25, 413–431. doi: 10.1108/IJOA-05-2016-1025

[ref3] Alutto J. A. Hrebiniak L. G. Alonso R. C. (1971). A study of differential socialization for members of one professional occupation. J. Health Soc. Behav. 12, 140–147. doi: 10.2307/2948521, PMID: 5088033

[ref4] Alvesson M. Kärreman D. (2006). “Professional service firms as collectivities: a cultural and Processual view,” in Professional service firms. 1st *Edn*. eds. Greenwood R. Suddaby R. (Amsterdam: Elsevier JAI), 203–230.

[ref5] Barrick M. R. Parks-Leduc L. (2019). Selection for fit. Annu. Rev. Organ. Psych. Organ. Behav. 6, 171–193. doi: 10.1146/annurev-orgpsych-012218-015028

[ref6] Baum M. Kabst R. (2013). How to attract applicants in the Atlantic versus the Asia-Pacific region? A cross-national analysis on China, India, Germany, and Hungary. J. World Bus. 48, 175–185. doi: 10.1016/j.jwb.2012.07.002

[ref7] Baum M. Kabst R. (2014). The effectiveness of recruitment advertisements and recruitment websites. Indirect and interactive effects on applicant attraction. Hum. Resour. Manag. 53, 353–378. doi: 10.1002/hrm.21571

[ref8] Becker G. S. (1994). Human capital: A theoretical and empirical analysis, with special reference to education. 3rd Edn. [Preprint]. Chicago, Ill. The Univ. of Chicago Press.

[ref9] Breaugh J. A. (2013). Employee recruitment. Annu. Rev. Psychol. 64, 389–416. doi: 10.1146/annurev-psych-113011-143757 23121331

[ref10] Byrne D. E. (1971). The attraction paradigm. New York: Acad. Press.

[ref11] Byrne D. E. (1997). An overview (and Underview) of research and theory within the attraction paradigm. J. Soc. Pers. Relat. 14, 417–431. doi: 10.1177/0265407597143008

[ref12] Cable D. M. Turban D. B. (2001). Establishing the dimensions, sources and value of job seekers’ employer knowledge during recruitment. Res. Pers. Hum. Resour. Manag. 20, 115–163. doi: 10.1016/S0742-7301(01)20002-4

[ref13] Chapman D. S. Uggerslev K. L. Carroll S. A. Piasentin K. A. Jones D. A. (2005). Applicant attraction to organizations and job choice. A meta-analytic review of the correlates of recruiting outcomes. J. Appl. Psychol. 90, 928–944. doi: 10.1037/0021-9010.90.5.928, PMID: 16162065

[ref14] Collins C. J. (2007). The interactive effects of recruitment practices and product awareness on job seekers’ employer knowledge and application behaviors. J. Appl. Psychol. 92, 180–190. doi: 10.1037/0021-9010.92.1.180, PMID: 17227159

[ref15] Collins C. J. Stevens C. K. (2002). The relationship between early recruitment-related activities and the application decisions of new labor-market entrants. A brand equity approach to recruitment. J. Appl. Psychol. 87, 1121–1133. doi: 10.1037/0021-9010.87.6.1121, PMID: 12558218

[ref16] Dalal D. K. Zickar M. J. (2012). Some common myths about centering predictor variables in moderated multiple regression and polynomial regression. Organ. Res. Methods 15, 339–362. doi: 10.1177/1094428111430540

[ref17] Delgado-Ballester E. Navarro A. Sicilia M. (2012). Revitalising brands through communication messages. The role of brand familiarity. Eur. J. Mark. 46, 31–51. doi: 10.1108/03090561211189220

[ref18] Dengler K. Stops M. Vicari B. (2016). “Occupation-specific matching efficiency”. IAB-Discussion PaperUR. Available at: https://www.econstor.eu/handle/10419/14615616/2016

[ref19] Dierdorff E. C. Morgeson F. P. (2007). Consensus in work role requirements: the influence of discrete occupational context on role expectations. J. Appl. Psychol. 92, 1228–1241. doi: 10.1037/0021-9010.92.5.1228, PMID: 17845082

[ref20] Dineen B. R. Ash S. R. Noe R. A. (2002). A web of applicant attraction. Person-organization fit in the context of web-based recruitment. J. Appl. Psychol. 87, 723–734. doi: 10.1037//0021-9010.87.4.723, PMID: 12184576

[ref21] Doverspike D. Taylor M. A. Shultz K. S. McKay P. F. (2000). Responding to the challenge of a changing workforce: recruiting nontraditional demographic groups. Public Personnel Manag. 29, 445–459. doi: 10.1177/009102600002900403

[ref22] Edwards J. R. (1994). The study of congruence in organizational behavior research: critique and a proposed alternative. Organ. Behav. Hum. Decis. Process. 58, 51–100. doi: 10.1006/obhd.1994.1029

[ref23] Ehrhart K. H. Ziegert J. C. (2005). Why are individuals attracted to organizations? J. Manag. 31, 901–919. doi: 10.1177/0149206305279759

[ref24] Elias Y. Nussinson R. Roccas S. (2018). Value-related goals and vocational choice: the effect of temporal distance. Eur. J. Soc. Psychol. 48, 93–99. doi: 10.1002/ejsp.2286

[ref25] Etzioni A. (1969). The semi-professions and their organization: Teachers, nurses, social workers. New York: Free Press.

[ref26] Evertz L. Süß S. (2017). The importance of individual differences for applicant attraction: a literature review and avenues for future research. Manag. Rev. Quarterly 67, 141–174. doi: 10.1007/s11301-017-0126-2

[ref27] Favero N. Bullock J. B. (2015). How (not) to solve the problem: an evaluation of scholarly responses to common source bias. Organ. Res. Methods 25, 285–308. doi: 10.1093/jopart/muu020

[ref28] Festinger L. (1957). A theory of cognitive dissonance. Stanford, California: Stanford University Press.

[ref29] Frederickson J. Rooney J. F. (1990). How the music occupation failed to become a profession. Int. Rev. Aesthet. Sociol. Music. 21:189. doi: 10.2307/837023

[ref30] Gibbons R. Waldman M. (2004). Task-specific human capital. Am. Econ. Rev. 94, 203–207. doi: 10.1257/0002828041301579

[ref31] Gilch P. M. Sieweke J. (2021). Recruiting digital talent: the strategic role of recruitment in organisations’ digital transformation. German J. Human Resource Manag. 35, 53–82. doi: 10.1177/2397002220952734

[ref32] Gioia D. A. Corley K. G. Hamilton A. L. (2013). Seeking qualitative rigor in inductive research. Notes on the Gioia methodology. Organ. Res. Methods 16, 15–31. doi: 10.1177/1094428112452151

[ref34] Grohmann B. (2009). Gender dimensions of brand personality. J. Mark. Res. 46, 105–119. doi: 10.1509/jmkr.46.1.105

[ref35] Harrison D. A. (2007). “Pitching fits in applied psychological research: making fit methods fit theory,” in Perspectives on organizational fit. eds. Ostroff C. Judge T. A. (New York: Erlbaum), 389–416.

[ref36] Highhouse S. Lievens F. Sinar E. F. (2003). Measuring attraction to organizations. Educ. Psychol. Meas. 63, 986–1001. doi: 10.1177/0013164403258403

[ref37] Hoerger M. (2010). Participant dropout as a function of survey length in internet-mediated university studies: implications for study design and voluntary participation in psychological research. Cyberpsychol. Behav. Soc. Netw. 13, 697–700. doi: 10.1089/cyber.2009.0445, PMID: 21142995 PMC4367493

[ref38] Hohenberger C. Grohs R. (2020). Old and exciting? Sport sponsorship effects on brand age and brand personality. Sport Manag. Rev. 23, 469–481. doi: 10.1016/j.smr.2019.05.002

[ref39] Holland J. L. (1997). Making vocational choices: a theory of vocational personalities and work environments. 3rd Edn. Odessa, FL: Psychological Assessment Resources.

[ref40] Ito J. K. Brotheridge C. M. McFarland K. (2013). Examining how preferences for employer branding attributes differ from entry to exit and how they relate to commitment, satisfaction, and retention. Career Dev. Int. 18, 732–752. doi: 10.1108/CDI-05-2013-0067

[ref41] Judge T. A. Cable D. M. (1997). Applicant personality, organizational culture, and organization attraction. Personnel psychology. Pers. Psychol. 50, 359–394. doi: 10.1111/J.1744-6570.1997.TB00912.X

[ref42] Kambourov G. Manovskii I. (2009). Occupational specificity of human capital. Int. Econ. Rev. 50, 63–115. doi: 10.1111/j.1468-2354.2008.00524.x

[ref43] Kerkmann C. (2019). “124.000 offene Stellen–Mangel an IT-Spezialisten nimmt dramatisch zu”. Available at: https://www.handelsblatt.com/technik/it-internet/bitkom-statistik-124-000-offene-stellen-mangel-an-it-spezialisten-nimmt-dramatisch-zu/25278122.html?ticket=ST-403554-wRNDBbSLKYkAMa5IbCq9-ap5 (Accessed March 18, 2020).

[ref44] Kim J. York K. M. Lim J.-S. (2011). The role of brands in recruitment: a mixed-brand strategy approach. Mark. Lett. 22, 165–179. doi: 10.1007/s11002-010-9119-9

[ref45] Koch I. Stahl C. (2017). “Lernen–Assoziationsbildung, Konditionierung und implizites Lernen,” in Allgemeine Psychologie. eds. Müsseler J. Rieger M. (Berlin, Heidelberg: Springer), 319–355.

[ref46] Kristof A. L. (1996). Person-organization fit: an integrative review of its conceptualizations, measurement, and implications. Pers. Psychol. 49, 1–49. doi: 10.1111/j.1744-6570.1996.tb01790.x

[ref47] Kristof-Brown A. Guay R. P. (2011). “Person–environment fit,” in APA handbook of industrial and organizational psychology, Vol 3: Maintaining, expanding, and contracting the organization. ed. Zedeck S. (Washington: American Psychological Association), 3–50.

[ref48] Kristof-Brown A. Zimmerman R. D. Johnson E. C. (2005). Consequences of Individuals’ fit at work: a meta-analysis of person-job, person-organization, person-group, and person-supervisor fit. Pers. Psychol. 58, 281–342. doi: 10.1111/j.1744-6570.2005.00672.x

[ref49] Kwon I. Meyersson Milgrom E. M. (2014). The significance of firm and occupation specific human capital for hiring and promotions. Labour Econ. 31, 162–173. doi: 10.1016/j.labeco.2014.07.003

[ref50] Lee C.-H. Hwang F.-M. Yeh Y.-C. (2013). The impact of publicity and subsequent intervention in recruitment advertising on job searching freshmen's attraction to an organization and job pursuit intention. J Appl Social Pyschol 43, 1–13. doi: 10.1111/j.1559-1816.2012.00975.x

[ref51] Leicht K. T. Lyman E. C. (2006). “Markets, institutions, and the crisis of professional practice,” in Professional service firms. 1st *Edn*. eds. Greenwood R. Suddaby R. (Amsterdam: Elsevier JAI), 17–44.

[ref52] Levy S. J. (1959). Symbols for Sale. Harv. Bus. Rev. 37, 117–124.

[ref53] Lievens F. Highhouse S. (2003). The relation of instrumental and symbolic attributes to a company’s attractiveness as an employer. Pers. Psychol. 56, 75–102. doi: 10.1111/j.1744-6570.2003.tb00144.x

[ref54] Lievens F. Slaughter J. E. (2016). Employer image and employer branding. What we know and what we need to know. Annu. Rev. Organ. Psych. Organ. Behav. 3, 407–440. doi: 10.1146/annurev-orgpsych-041015-062501

[ref55] Lievens F. van Hoye G. Schreurs B. (2005). Examining the relationship between employer knowledge dimensions and organizational attractiveness. An application in a military context. J. Occup. Organ. Psychol. 78, 553–572. doi: 10.1348/09631790X26688

[ref56] Lin N. (2001). Social capital: A theory of social structure and action. 1. 1st Edn. Cambridge: Cambridge Univ. Press.

[ref57] Mason E. S. Mudrack P. E. (1996). Gender and ethical orientation: a test of gender and occupational socialization theories. J. Bus. Ethics 15, 599–604. doi: 10.1007/BF00411793

[ref58] McAlister A. R. Cornwell T. B. (2010). Children's brand symbolism understanding: links to theory of mind and executive functioning. Psychol. Mark. 27, 203–228. doi: 10.1002/mar.20328

[ref59] McDonald S. (2011). What you know or who you know? Occupation-specific work experience and job matching through social networks. Soc. Sci. Res. 40, 1664–1675. doi: 10.1016/j.ssresearch.2011.06.003

[ref60] Miller S. E. (2013). Professional socialization: a bridge between the explicit and implicit curricula. J. Soc. Work. Educ. 49, 368–386. doi: 10.1080/10437797.2013.796773

[ref61] Montoya R. M. Horton R. S. Kirchner J. (2008). Is actual similarity necessary for attraction? A meta-analysis of actual and perceived similarity. J. Soc. Pers. Relat. 25, 889–922. doi: 10.1177/0265407508096700

[ref62] Newman D. A. Lyon J. S. (2009). Recruitment efforts to reduce adverse impact. Targeted recruiting for personality, cognitive ability, and diversity. J. Appl. Psychol. 94, 298–317. doi: 10.1037/a0013472, PMID: 19271792

[ref63] Nordin M. Persson I. Rooth D.-O. (2010). Education–occupation mismatch: is there an income penalty? Econ. Educ. Rev. 29, 1047–1059. doi: 10.1016/j.econedurev.2010.05.005

[ref64] Paroway (2020). “Volkswagen says it is changing into a software-driven tech business”. Available at: https://www.greencarcongress.com/2020/03/20200319-vw.html (Accessed October 22, 2021).

[ref65] Pernkopf K. Latzke M. Mayrhofer W. (2021). Effects of mixed signals on employer attractiveness: a mixed-method study based on signalling and convention theory. Hum. Resour. Manag. J. 31, 392–413. doi: 10.1111/1748-8583.12313

[ref66] Podsakoff P. M. MacKenzie S. B. Lee J.-Y. Podsakoff N. P. (2003). Common method biases in behavioral research. A critical review of the literature and recommended remedies. J. Appl. Psychol. 88, 879–903. doi: 10.1037/0021-9010.88.5.879, PMID: 14516251

[ref67] Ritz A. Waldner C. (2011). Competing for future leaders. A study of attractiveness of public sector organizations to potential job applicants. Rev. Public Personnel Admin. 31, 291–316. doi: 10.1177/0734371X11408703

[ref500] Rynes S. L. (1991). “Recruitment, job choice, and post-hire consequences,” in Handbook of Industrial and Organizational Psychology. 2nd *Edn*. Palo Alto, CA: Consulting Psychologists Press, 399–444.

[ref68] Schreurs B. Druart C. Proost K. Witte K. De. (2009). Symbolic attributes and organizational attractiveness. The moderating effects of applicant personality. Int. J. Sel. Assess. 17, 35–46. doi: 10.1111/j.1468-2389.2009.00449.x

[ref69] Statista Research Department (2021). Female STEM academics and STEM professionals among all employed STEM academics and STEM professionals in Germany from 2011 to 2018.

[ref70] Siemsen E. Roth A. Oliveira P. (2010). Common method bias in regression models with linear, quadratic, and interaction effects. Organ. Res. Methods 13, 456–476. doi: 10.1177/1094428109351241

[ref71] Simmering M. J. Fuller C. M. Richardson H. A. Ocal Y. Atinc G. M. (2015). Marker variable choice, reporting, and interpretation in the detection of common method variance. A review and demonstration. Organ. Res. Methods 18, 473–511. doi: 10.1177/1094428114560023

[ref72] Skorikov V. B. Vondracek F. W. (2011). “Occupational identity,” in Handbook of identity theory and research. ed. Schwartz S. J. (New York: Springer), 693–714.

[ref73] Su R. Murdock C. Rounds J. (2015). “Person-environment fit,” in APA handbook of career intervention, volume 1: Foundations. eds. Hartung P. J. Savickas M. Walsh W. B. (Washington, D.C: American Psychological Association), 81–98.

[ref74] Sullivan P. (2010). Empirical evidence on occupation and industry specific human capital. Labour Econ. 17, 567–580. doi: 10.1016/j.labeco.2009.11.003, PMID: 20526448 PMC2879094

[ref75] Super D. E. (1953). A theory of vocational development. Am. Psychol. 8, 185–190. doi: 10.1037/h0056046

[ref76] Thoms P. Goodrich J. Chinn S. J. Howard G. (2004). Designing personable and informative job recruiting web sites. Testing the effect of the design on attractiveness and intent to apply. PR 95, 1031–1042. doi: 10.2466/PR0.95.7.1031-1042 15217067

[ref77] Tsai Y.-H. Lin C.-P. Ma H.-C. Wang R.-T. (2015). Modeling corporate social performance and job pursuit intention. Forecasting the job change of professionals in technology industry. Technol. Forecast. Soc. Chang. 99, 14–21. doi: 10.1016/j.techfore.2015.06.026

[ref78] Tsai W.-C. Yang I. W.-F. (2010). Does image matter to different job applicants? The influences of corporate image and applicant individual differences on organizational attractiveness. Int. J. Sel. Assess. 18, 48–63. doi: 10.1111/j.1468-2389.2010.00488.x

[ref79] Turban D. B. (2001). Organizational attractiveness as an employer on college campuses. An examination of the applicant population. J. Vocat. Behav. 58, 293–312. doi: 10.1006/jvbe.2000.1765

[ref80] Überschaer A. Baum M. Bietz B.-T. Kabst R. (2016). The contingencies of person-organization fit perceptions. J. Managerial Psych 31, 1021–1039. doi: 10.1108/JMP-09-2014-0266

[ref81] Uggerslev K. L. Fassina N. E. Kraichy D. (2012). Recruiting through the stages. A meta-analytic test of predictors of applicant attraction at different stages of the recruiting process. Pers. Psychol. 65, 597–660. doi: 10.1111/j.1744-6570.2012.01254.x

[ref82] van Hoye G. Bas T. Cromheecke S. Lievens F. (2013). The instrumental and symbolic dimensions of organisations’ image as an employer. A large-scale field study on employer branding in Turkey. Appl. Psychol. 62, 543–557. doi: 10.1111/j.1464-0597.2012.00495.x

[ref83] van Vianen A. E. (2018). Person–Environment Fit: a Review of Its Basic Tenets. Annu. Rev. Organ. Psych. Organ. Behav. 5, 75–101. doi: 10.1146/annurev-orgpsych-032117-104702

[ref84] Verquer M. L. Beehr T. A. Wagner S. H. (2003). A meta-analysis of relations between person–organization fit and work attitudes. J. Vocat. Behav. 63, 473–489. doi: 10.1016/S0001-8791(02)00036-2

[ref85] von Nordenflycht A. (2010). What is a professional service firm? Toward a theory and taxonomy of knowledge-intensive firms. Amrudh 35, 155–174. doi: 10.5465/amr.35.1.zok155

[ref86] Walker H. J. Feild H. S. Bernerth J. B. Becton J. B. (2012). Diversity cues on recruitment websites. Investigating the effects on job seekers' information processing. J. Appl. Psychol. 97, 214–224. doi: 10.1037/a0025847, PMID: 22004220

[ref87] Williams L. J. Hartman N. Cavazotte F. (2010). Method variance and marker variables. A review and comprehensive CFA marker technique. Organ. Res. Methods 13, 477–514. doi: 10.1177/1094428110366036

[ref88] Witzel A. Reiter H. (2012). The problem-Centred interview: Principles and practice. 1 Oliver’s yard, 55 City road, London EC1Y 1SP United Kingdom: SAGE Publications Ltd..

[ref89] Yu K. Y. T. (2014). Person–organization fit effects on organizational attraction. A test of an expectations-based model. Organ. Behav. Hum. Decis. Process. 124, 75–94. doi: 10.1016/j.obhdp.2013.12.005

[ref90] Yu K. Y. T. Davis H. M. (2019). Integrating job search behavior into the study of job seekers’ employer knowledge and organizational attraction. Int. J. Hum. Resour. Manag. 30, 1448–1476. doi: 10.1080/09585192.2017.1288152

